# Multi-omics network analysis reveals distinct stages in the human aging progression in epidermal tissue

**DOI:** 10.18632/aging.103499

**Published:** 2020-06-18

**Authors:** Nicholas Holzscheck, Jörn Söhle, Boris Kristof, Elke Grönniger, Stefan Gallinat, Horst Wenck, Marc Winnefeld, Cassandra Falckenhayn, Lars Kaderali

**Affiliations:** 1 Front End Innovation, Beiersdorf AG, Hamburg, Germany; 2 Institute for Bioinformatics, University Medicine Greifswald, Greifswald, Germany

**Keywords:** multi-omics, biological age, aging phases, hallmarks of aging, transcriptional noise

## Abstract

In recent years, reports of non-linear regulations in age- and longevity-associated biological processes have been accumulating. Inspired by methodological advances in precision medicine involving the integrative analysis of multi-omics data, we sought to investigate the potential of multi-omics integration to identify distinct stages in the aging progression from *ex vivo* human skin tissue. For this we generated transcriptome and methylome profiling data from suction blister lesions of female subjects between 21 and 76 years, which were integrated using a network fusion approach. Unsupervised cluster analysis on the combined network identified four distinct subgroupings exhibiting a significant age-association. As indicated by DNAm age analysis and Hallmark of Aging enrichment signals, the stages captured the biological aging state more clearly than a mere grouping by chronological age and could further be recovered in a longitudinal validation cohort with high stability. Characterization of the biological processes driving the phases using machine learning enabled a data-driven reconstruction of the order of Hallmark of Aging manifestation. Finally, we investigated non-linearities in the mid-life aging progression captured by the aging phases and identified a far-reaching non-linear increase in transcriptional noise in the pathway landscape in the transition from mid- to late-life.

## INTRODUCTION

Biological age represents the main risk factor for most chronic human pathologies, which is why therapies slowing the aging progression and postponing the onset of age-driven disease manifestation have frequently been suggested as major interventions to improve human health span. Chronological age has long been utilized as a proxy for biological aging state, in recent years however, the heterogeneity of biological aging rates for individuals of the same chronological age has become increasingly apparent. The most prominent example for this decoupling has probably been delivered in the wake of the discovery of the “epigenetic clock” in both mouse and human tissues [1–7], which revealed accelerated aging rates associated with various disease states and all-cause mortality [8–11], and is measured by DNA methylation state.

The notion of aging being a continuous process meanwhile remained. Lately though, this view has been questioned by reports on non-linearity and discontinuities in biological processes associated with aging and longevity. Early indications included the identification of two distinguishable phases in the aging progression of *Drosophila melanogaster* [12]. The transition to the second aging phase, marked by decreased motor activity and heightened inflammation, was accompanied by an exponentially increased mortality risk. Remarkably, this 2-phased model was able to reproduce a variety of experimental longevity curves [12]. More recently, evidence of the existence of non-linear switches, capable of extending model animal lifespan *in vivo*, has been presented concerning mitochondrial function, further implicating discontinuous biological processes in aging [13]. Not long ago now, the report of a mid-life switch involving a longevity-associated signaling pathway in aging human muscle and brain tissue was published [14]. Using gene and long non-coding RNA expression profiling, the authors observed that an age-related IGF-1/PI3K/mTOR-related RNA response signature was essentially lost with the start of the sixth decade of life. The report provides compelling evidence that discontinuous processes might be a previously overlooked feature of human aging as well and indicate that the progression of biological aging on a molecular level might be even more intricately regulated and complex than previously assumed.

The different biological processes driving aging meanwhile are manifold. The Hallmarks of Aging [15] provide a description of nine common denominators of aging in different tissues and organisms, attempting a categorization of various biological pathways into conceptual cornerstones of aging. Based on extensive literature review, the authors not only grouped, but also postulated the order of emergence for the different hallmarks. While the theoretical depiction of these hallmarks is detailed and comprehensive, a data-driven characterization of their importance to the aging phenotype and the actual disentanglement of the timely order of their occurrence in *in vivo* human tissue have remained elusive.

Recent years have seen a continuous decline in costs for genome-wide analyses, leading to an increasing feasibility of multi-omics profiling studies. Simultaneously monitoring multiple different omics levels in a living system holds great promises in generating a holistic understanding of phenotypical manifestations and might prove beneficial for aging research, as it has for the medical sciences. However, the integration of multi-omics data also brings tremendous novel statistical and computational challenges. These are related to the properties of many omics datasets, which include high dimensionality with often low sample counts, differing scales and distributions of measurements, as well as platform specific bias and technical noise [16]. In order to tackle these challenges and to uncover complementary information from multi-omics data, an increasing number of algorithmic approaches have been developed in the past years [17]. Network based methods such as similarity network fusion (SNF) offer an elegant solution to the problem, by transferring the feature-patient data for each dataset into featureless patient-patient space before their integration [18]. From every dataset a similarity network is created with patients represented as nodes and similarities between patients as edges. The separate networks are then integrated through an iterative fusion algorithm, which strengthens edges present in several data views, and finally converges into a fused network. This final network incorporates similarities from all omics data views and can be used for downstream analyses such as subtype identification through clustering.

In an effort to further explore the discontinuities in the aging progression using multi-omics methodology, we generated gene expression and DNA methylation data from *ex vivo* samples of aging skin. Skin represents an extraordinarily well-suited tissue for studying aging, owing to its well-documented aging phenotype and the ease of sampling using well-established non- or minimally invasive procedures. Using similarity network fusion, we integrated and clustered the multi-omics data to identify discrete stages along the aging progression. We validated the latent stages using DNA methylation age, the detection of Hallmark of Aging signals, and using a longitudinal validation cohort. Finally, we deployed machine learning to elucidate the order of Hallmark of Aging manifestation throughout the aging phases, and characterized the phases regarding pathway importance, which subsequently revealed a distinctly non-linear decrease in pathway enrichment at the mid- to late-life transition from aging phase 3 to phase 4.

## RESULTS

### Identification of latent age-associated molecular stages

**Figure 1:**
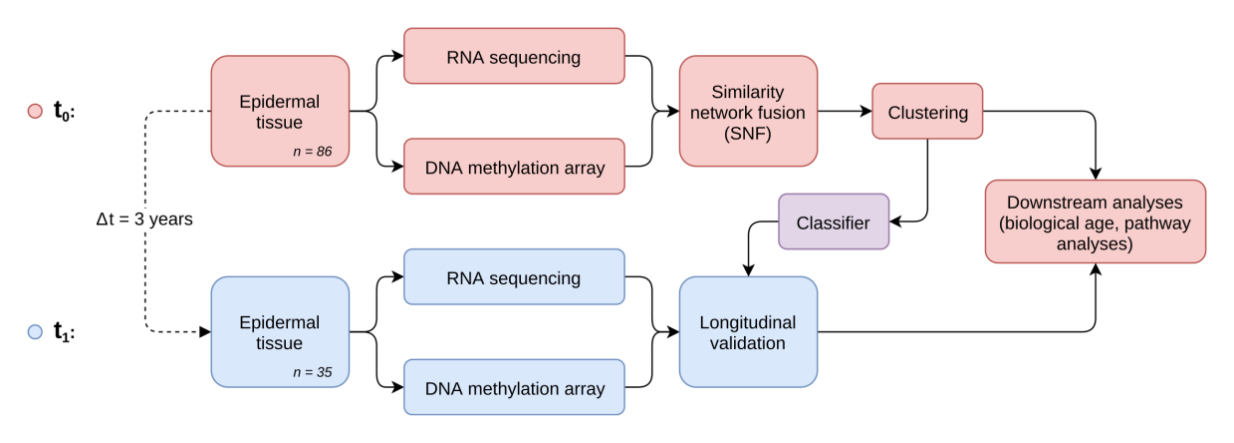
**Study and analysis setup.** Workflow diagram depicting the two-stage longitudinal study setup and the main steps of multi-omics data generation, integration and analysis.

**Figure 2:**
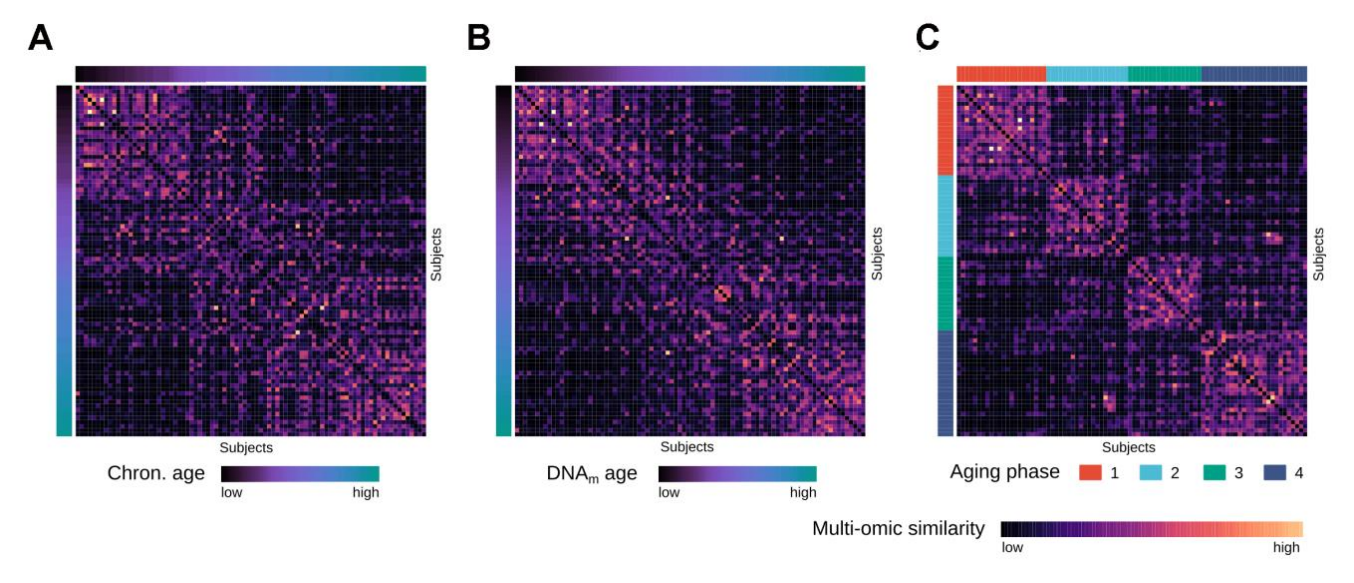
**Multi-omics similarity between subjects in the integrated network.** (**A**) Heatmap visualization showing similarities between subjects in the fused multi-omics similarity network generated from gene expression and methylation data, with subjects ordered by increasing chronological age. (**B**) Same heatmap visualization of multi-omics similarity as in (**A**), with subjects ordered by increasing DNAm age. (**C**) Same heatmap visualization of multi-omics similarity as in (**A**) and (**B**), with the subjects ordered by the identified aging phases.

**Figure 3:**
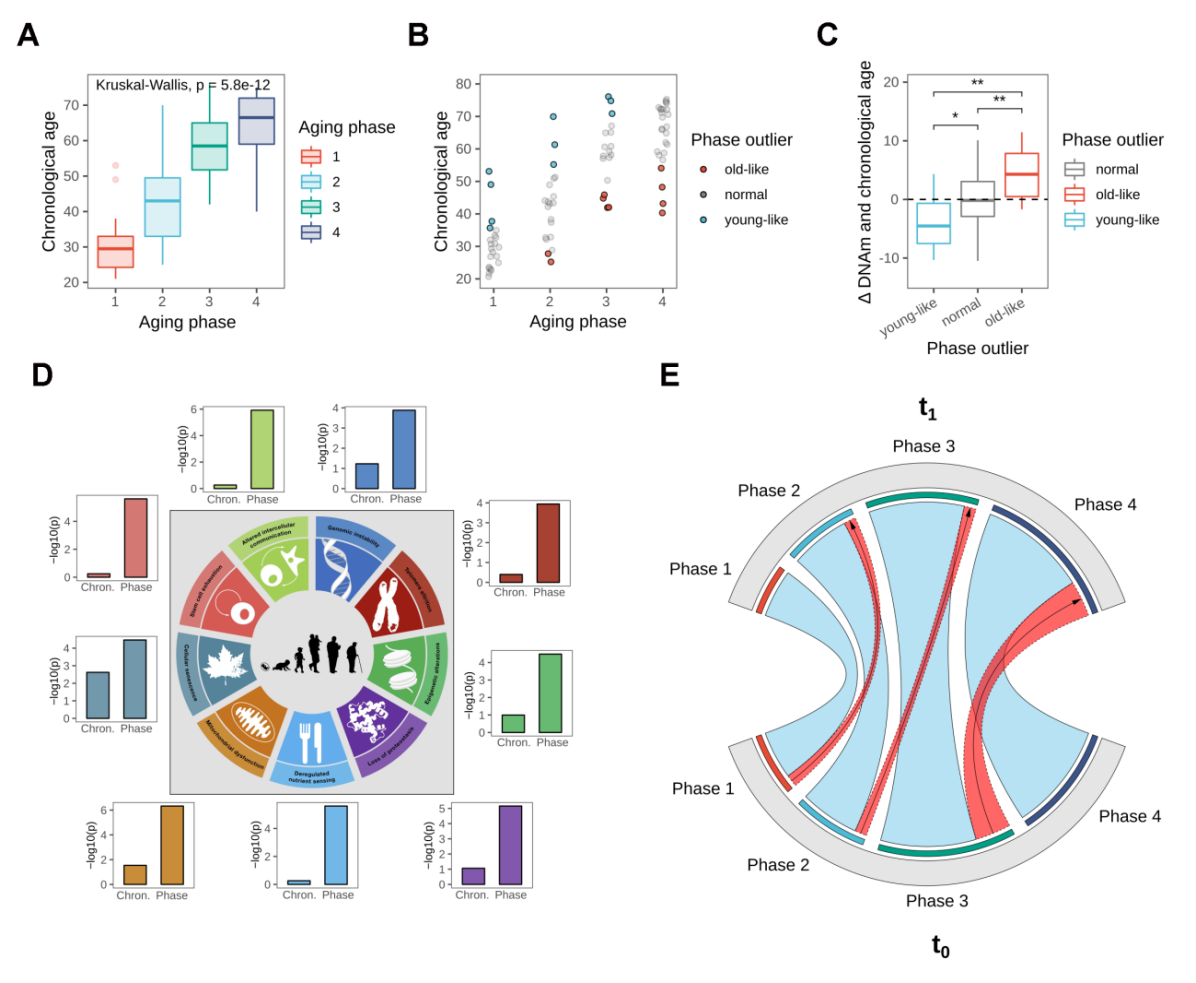
**Biological age validation of the identified phases.** (**A**) Boxplot showing chronological age distributions among the four identified aging phases. (**B**) Chronological age outliers among the aging phases, denoted as “old-like” for subjects that appeared to prematurely cluster into a higher aging phase, and “young-like” for subjects that were classified into a lower aging phase relative to their chronological age. (**C**) Boxplot showing the deviation of DNAm from chronological age based on aging phase outlier status, revealing a divergence in DNAm aging rate for aging phase outliers. Statistical significance determined using pairwise T-tests. (**D**) Hallmark of Aging signal strengths in gene expression data, comparing chronological age groups to the biological aging phases. Shown are the adjusted p-values from Anova comparisons, testing the segregation of the groupings among gene set enrichment scores. Figure adapted from the original Hallmark of Aging publication [15]. (**E**) Longitudinal validation after three-year period. The chord diagram shows aging phase classification of re-invited subjects at both time points, with phase transitions highlighted in red.

To identify distinct stages in aging skin tissue, we examined 86 female subjects between 21 and 76 years. Subjects were chosen so that all ages were represented evenly and were required to be in good health. From each subject we sampled epidermal tissue from the subject’s volar forearms via the suction blister method. 31 of the original subjects were further re-invited for a longitudinal second measurement, which took place three years later (Figure 1). From the epidermal samples we generated gene expression and DNA methylation data, which were computationally integrated using the similarity network fusion approach. The resulting network, incorporating information from both transcriptomes and methylomes, was then used to identify hidden subtypes via unsupervised spectral clustering. The clustering revealed four distinct subgroups in our data with roughly equal sizes of 22 (cluster 1), 20 (cluster 2), 18 (cluster 3) and 26 (cluster 4) subjects, that captured the multi-omics similarity structure between the samples more clearly than either chronological or DNA methylation (DNAm) age (Figure 2A-2C). Association analysis to subject metadata showed that the clusters were significantly associated with chronological age (p = 5.8e-12, Figure 3A), whilst not being confounded by BMI (p = 0.71, Supplementary Figure 1A).

### Latent stages associate more strongly with DNAm age rather than chronological age

As the unsupervised and purely data-driven clustering had identified groupings with strong association to chronological age, we explored the possibility that the clusters might capture hidden stages in the aging progression. We hypothesized that if this were so, the groupings ought to be more strongly associated with the actual biological aging state of our subjects, rather than their chronological age. To test this, DNAm ages of all subjects were calculated as previously described [19], to serve as a proxy measure for biological age (Supplementary Figure 1B). The comparison revealed a stronger association of the identified stages to DNAm age (p = 3.9e-13) as opposed to chronological age (p = 5.2e-12), strengthening the hypothesis that the clusters captured multi-omics aging stages.

### Aging phase outliers are also biological age outliers in the sense of DNAm age

As subjects within the phases still presented considerable variation in chronological age, and the most proven approximation to biological age available to date is through the use of DNAm age, we further explored if the chronological outliers in the different aging phases were also outliers in the sense of DNAm age. We defined individuals as outliers for every aging phase (Figure 3B) if their chronological age either exceeded the 3^rd^ quartile by at least one third of the interquartile range (“young-like”) or subceeded the 1^st^ quartile by said amount (“old-like”). Analysis of the deviation of chronological to DNAm ages revealed that phase outliers were indeed biologically significantly younger (mean/median = -3.8/-4.5 y) or older (mean/median = +4.2/+4.3 y) than average and also than each other respectively (Figure 3C). Association testing using age-adjusted logistic regression models further revealed that subjects assigned to the “old-like” group were also significantly more likely to have reported frequent sun bathing in the questionnaires (p = 0.0336), delivering evidence of photoaging factoring into aging phase assignment. We further repeated the analysis using age estimates from a transcriptomic clock [19], which on average showed lower accuracy than its DNAm counterpart (Supplementary Figure 1C), in concordance with previous reports [19]. Association testing of phase outlier status to transcriptomic age revealed the same trends observed with DNAm age, albeit in this case without reaching statistical significance (Supplementary Figure 1D). Notably, the correlation of the two biological age markers was lower than their respective correlations to chronological age (Supplementary Figure 1E), indicating that the clocks capture at least partly independent features of aging, again underlining the importance of multi-omics approaches for aging research.

### Aging phases show improved detection of Hallmark of Aging signals

The Hallmarks of Aging (HoA) describe nine main biological motives and processes that are believed to be driving the aging progression. We hypothesized that if the identified aging phases captured stages of biological aging, this ought to be reflected in gene expression patterns related to the known aging cornerstone processes, as summarized by the HoA. We therefore generated lists of genes involved in each of the nine HoA, by selecting GO and Reactome pathways which captured the essence of the respective hallmarks and combining them to novel gene sets (Supplementary Figure 2A). We then used the sets to test if the aging phases allowed better detection of HoA-enrichment signals than chronological age groups. ANOVA analyses using gene set enrichment scores indeed showed stronger discrimination based on aging stage for all HoA gene sets (Figure 3D). This further extends the evidence of the phases capturing biological multi-omics age to the level of gene expression.

### Longitudinal validation of aging phases over three-year period

To assess if the phases could be longitudinally reproduced, 31 subjects from the original cohort were re-invited three years later for a second measurement. To assess the aging phase of the new samples, a random forest classification model was built on both expression and methylation features from data of the original cohort. The classifier demonstrated high accuracy (AUC = 0.95) in discriminating between the four phases in repeated cross-validation on the original data (Supplementary Figure 2B) and was subsequently used to predict the aging stages of all subjects at the second time point. For most subjects the aging phase did not change within the 3-year-period, indicating high stability of the identified groupings (Figure 3E). This finding is not unexpected, considering the time span of only three years past since original sampling, relative to the much larger average phase windows with a standard deviation of between 8.2-11.5 chronological years. Nonetheless five subjects could be observed migrating from one aging phase to another, all of them transitioning naturally along the age gradient to the next phase (Figure 3E). Notably, four of these five subjects were previously classified as chronological outliers at the upper end of their age phase (Supplementary Figure 2C).

### Data-driven ranking of the Hallmarks of Aging along the phases reveals distinct succession patterns

**Figure 4:**
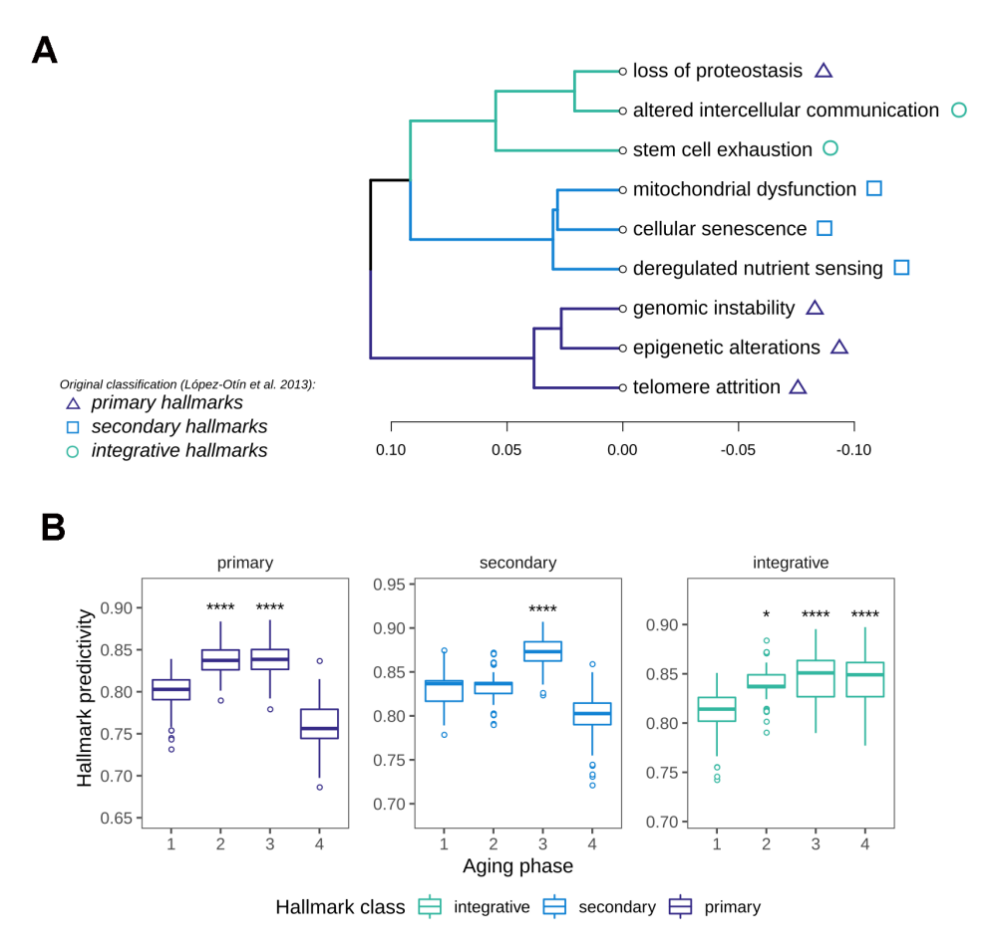
**Characterization of Hallmark of Aging predictivity within the aging phases.** (**A**) Hierarchical clustering of the nine Hallmarks of Aging based on their gene set predictivity analysis along the four aging phases. Predictivity was determined using cross-validated random forest classifiers, trained to distinguish each of the aging phases from the others. (**B**) Predictivity of the Hallmark of Aging gene sets along the four aging phases, grouped into primary, secondary and integrative hallmarks. Statistical testing was performed using one-sided Wilcoxon tests. All predictivity scores were derived from 100 permutations.

In order to identify the most important biological motives driving the aging phases, we resorted to the use of machine learning models. For this we implemented a method based on classifiers that learn to distinguish between aging phases, whilst taking advantage of biological pathway information in the training process. The workflow consisted of a stepwise training of classifiers only on subsets of genes annotated to pathways to predict aging phase from gene expression. By restricting the training to these genes, the cross-validated accuracies of these classifiers allow the assessment of how well a given gene set enables the differentiation between aging phases, thus resulting in a score for each pathway’s relevance. This score can intuitively be interpreted as a measure of how important or predictive a gene set is to the grouping of interest. In order to derive a data-driven ranking of the Hallmarks of Aging along the aging phases, we performed this pathway predictivity analysis using the aforementioned HoA gene sets, calculating 100 permutations for each pathway model. The predictivity scores revealed a clear patterning of the HoA along the four phases that allowed a grouping of the hallmarks using hierarchical clustering (Figure 4A). Strikingly, the hallmarks clustered almost in the exact constellations postulated in their original description [15], namely into primary hallmarks (genomic instability, telomere attrition, epigenetic alterations and originally loss of proteostasis), antagonistic or secondary hallmarks (cellular senescence, deregulated nutrient sensing and mitochondrial dysfunction) and integrative hallmarks (altered intercellular communication and stem cell exhaustion). Our analysis did however reveal a divergence in the classification of the proteostasis-hallmark, which clustered more strongly with the group of integrative hallmarks. Examination of the HoA predictivity patterns based on the newly generated classification revealed that the predictivity peaks for the respective hallmark classes extracted through our analysis (Figure 4B) also precisely match the temporal manifestation sequence postulated in the original description of the hallmarks as well [15]: Namely, primary hallmarks peaked in aging phases 2 and 3, followed by a sharp drop in predictivity thereafter. Meanwhile the importance of the secondary hallmarks increased notably in aging phase 3. The integrative hallmarks, postulated to be emerging as a consequence of primary and secondary manifestation, increased slowly along the phases, while peaking in the late aging phases 3 and 4, again in concordance with the original postulation [15]. To our knowledge this is the first data-driven validation of the overarching sequence in which these cornerstones of aging manifest in human tissue.

### Pathway predictivity analysis reveals a distinctly non-linear loss in pathway predictivity in old-age

**Figure 5:**
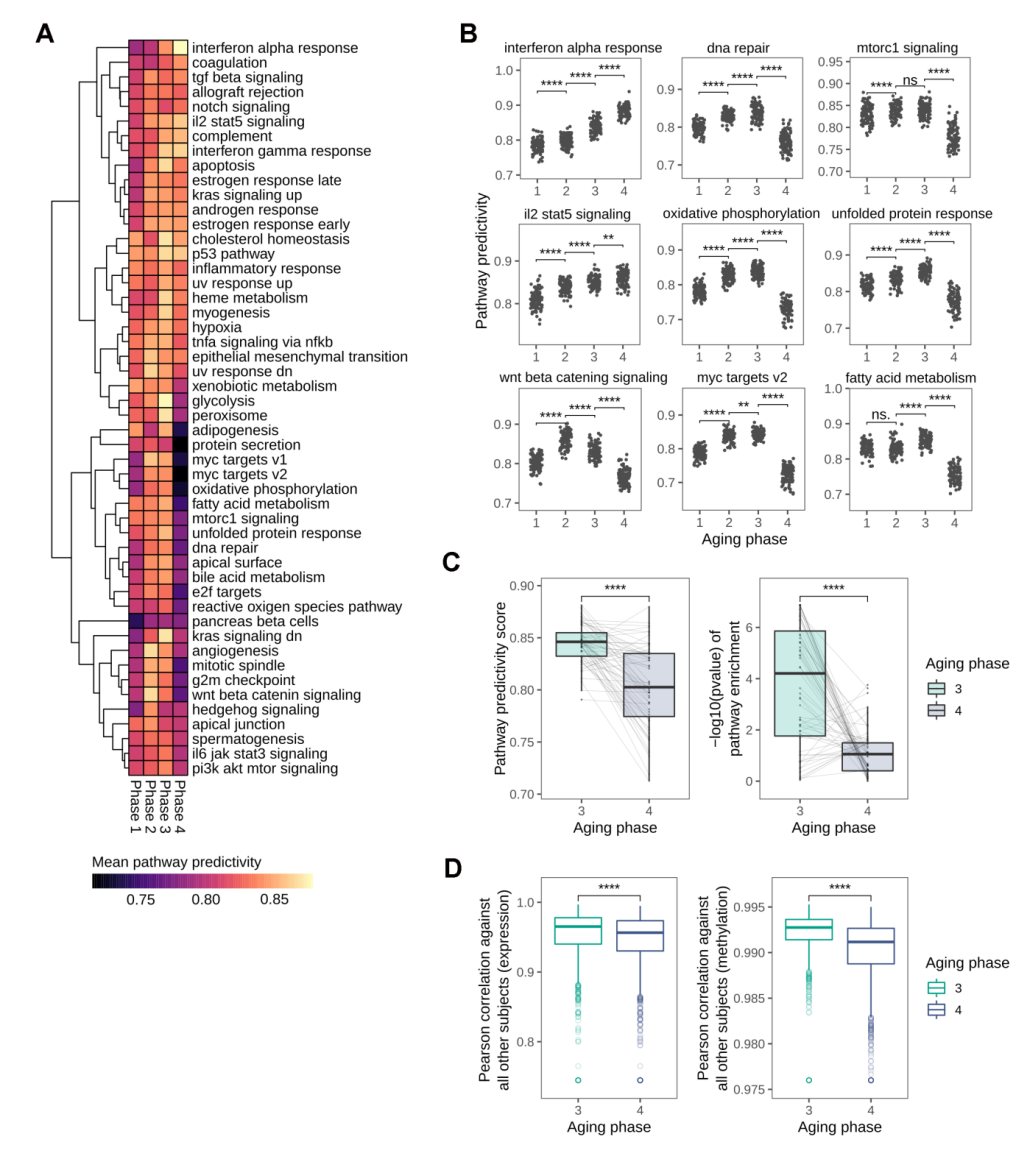
**Global loss in pathway predictivity in the transition from mid- to late-life.** (**A**) Heatmap showing the changes in pathway predictivity along the identified aging phases. The predictivities shown are the average predictivities calculated from 100 permutations for every pathway. (**B**) Scatterplots visualizing the changes in predictivity along the aging phases for selected pathways, several of which show distinctly non-linear patterns. (**C**) Overall loss in pathway predictivity observed in the transition from aging phase 3 to phase 4 is also detectable using gene set enrichment analysis. (**D**) Pairwise Pearson correlation between all subjects based on transcriptional and DNA methylation patterns.

As our analyses on the succession of the HoA already indicated a distinct shift in predictivities towards phase 4 of the identified aging stages, and in light of the recent publication of a sharp loss of signature identity for longevity-associated mTOR-signaling in human tissue around 60 years [14], we decided to expand our pathway analysis and explore the mid-to-late-life transition in more detail. For this we utilized the hallmark gene sets defined by the Broad Institute into the analysis, a set of conserved and highly refined gene sets created to improve pathway inference by reducing variance and gene overlap [20]. The predictivity analysis using these gene sets revealed a number of pathways that showed distinctly non-linear predictivity patterns along the four aging phases (Figure 5A). Notably, the most prominent global pattern was a sharp loss in predictivity observed in the transition from aging phase 3 to aging phase 4 in many pathways. In line with the loss of predictivity in nutrient sensing signaling hallmark observed in the HoA analysis and recent reports [14], mTOR-related signaling was among the pathways undergoing this distinct transition in the transition from phase 3 to phase 4 (Figure 5B), which, biological age outliers aside, matches the chronological age threshold of 60 identified in recent reports [14]. Other pathways exhibiting this pattern included oxidative phosphorylation and fatty acid metabolism, and notably also DNA repair pathways (Figure 5B). Exceptions from this trend included interferon and interleukin signaling, which increased steadily in predictivity along the phases, in line with the inflammaging theory of aging [21, 22], and the previously observed patterns in the HoA analysis. Apart from these exceptions, statistical analysis of all pathway predictivity signals between aging phases 3 and 4 still revealed a significant decrease in pathway predictivity, that is replicated using gene set enrichment analysis, also showing a distinct loss in pathway enrichment in transition to phase 4 (Figure 5C). As this finding potentially points to an increase in transcriptional noise, we investigated whether there was a change in the transcriptional similarity between subjects with transition into aging phase 4. For this we calculated pairwise correlations between the full transcriptomes of all subjects. In line with the results from the pathway analysis, we observed a significant drop in transcriptional similarity in the transition from phase 3 to phase 4. Notably, a similar effect can be observed in the methylation data, where a concomitant decrease in correlation between methylation profiles is observed (Figure 5D).

Together these findings indicate a distinctly non-linear increase in biological noise in the transition from mid-to-late-life, likely to contribute to the deterioration of human tissue function in old age.

## DISCUSSION

In this study we applied network based multi-omics integration to investigate non-linearity in the *in vivo* human aging progression. Similarity network fusion has so far mostly seen use in cancer research, in other fields there have only been rare applications of this methodology so far. To the best of our knowledge this publication represents the first documented use of a network based multi-omics integration and cluster analysis in the context of aging. The four aging stages that we identified in the integrated similarity network were more strongly associated with measures for biological age as opposed to chronological age, demonstrating the use of unsupervised integration and clustering in approximating biological aging and elucidating discrete stages from multi-omics data in the process.

To characterize the aging stages, we turned to the conceptual cornerstones that are believed to drive organismal aging, the so-called Hallmarks of Aging. For this we devised a novel approach to rank the gene sets according to their importance for the aging phases using machine learning methodology. The approach allowed us to validate the originally proposed classification of the hallmarks in a data-driven way and further to elucidate the order of their occurrence from the molecular data. The overall concordance of the data-driven reconstruction of the order of hallmark manifestation to the postulated sequence of succession is striking. All hallmarks clustered according to the proposed classification into primary, antagonistic and integrative hallmarks, with the only notable exception being the loss of proteostasis hallmark, which somewhat deviated from its postulated order. Based on this, a reclassification of this hallmark might be advisable. To resolve this, further investigations greatly expanding the width of studied tissues will be required though. The order of succession reconstructed from our data matches the proposed order of primary, antagonistic or secondary and integrative hallmarks almost perfectly. The earliest recorded peak is observed for the primary hallmarks in aging phase 2 and is followed by a significant increase in predictivity in phase 3 for the secondary hallmarks. This also includes an increased importance of mitochondrial processes around mid-life, a finding that is especially interesting in light of recent reports that a mid-life intervention alleviating mitochondrial dysfunction is sufficient to significantly increase health span in model animals [13]. The integrative hallmarks and in particular altered intercellular communication, a hallmark strongly based on immune and inflammatory signaling pathways, slowly increases in predictivity and peaks in the late aging phases, supporting the inflammaging theory of aging [22, 23]. Inflammaging describes the process by which immunosenescence and thus reduced ability to deal with stressors lead to a chronic low grade proinflammatory state in aged tissue, in turn deregulating the immune response and increasing vulnerability to pathologies with inflammatory genesis or progression.

The transitions to phase 3 and phase 4 were marked by various changes in the predictivity ranking of the hallmarks for each phase, indicating substantial rearrangements in the biological processes around mid-life. We investigated these transitions further by expanding our analysis to a wider range of conserved biological pathways. The analysis results showed a marked and global loss in pathway predictivity and pathway enrichment in the transition out of phase 3, indicative of an increase in transcriptional noise in aging phase 4. Pairwise correlation of all subjects further confirmed a significant deterioration of both transcriptomic and epigenetic patterning in the passage from aging phase 3 to aging phase 4. Chronological outliers aside, the onset of phase 3 exactly matches the median age at onset of menopause, which is around 51 years for Caucasian women in industrialized countries [24–26], a period that is indeed known to have substantive effects on the biology of the female body through wide-reaching hormonal adjustments. This is particularly interesting as the menopausal transition has been shown to accelerate biological aging based on large-scale analyses of blood derived DNAm age [27]. This finding has thus far lacked mechanistic explanation, the significant mid-life shift and loss in predictivity we observed in the pathway landscape in the transition from phase 3 to post-menopausal phase 4 might be a connected phenomenon and serve as starting point for further investigations into this matter. Notably, one of the hallmarks suffering a sharp loss in predictivity in this phase is the epigenetic alterations hallmark, linking the loss in transcriptional pathway state to an epigenetic age acceleration. Meanwhile, in a skin-specific context, the reports of accelerated skin aging following menopause are also manifold [28] and might equally be connected to our findings. A direct coupling between the identified aging phases 3 and 4 and the menopausal transition might explain yet another interesting epidemiological finding: the fact that higher age at onset of natural menopause has frequently been associated with greater remaining life expectancy and reduced all-cause mortality [26, 29–31]. Considering menopause as a distinct stage in the natural aging progression would allow the interpretation that women entering it later (at a higher chronological age) are biologically younger or “young-like” in the sense of the outlier classification proposed earlier (Figure 3B and 3C). The observed greater remaining life expectancy would then present itself as a plausible consequence of their lower biological age entering menopause.

One of the pathways that notably lost predictivity at the beginning of aging phase 4 was PI3K-mTOR-signaling, a known longevity-associated pathway, whose regulation has recently been reported to be largely lost around the chronological age of 60 [14]. Among the other pathways affected by a similar decrease in pathway enrichment were also DNA repair pathways. This might present a finding with significant impact to health in aging phase 4 onwards, as these pathways are crucial for cancer protection, and mutations and dysregulation in these pathways have been identified numerous times as drivers of tumorigenesis. The observed loss in pathway enrichment in the transition to phase 4 could be a worrying sign of decreased safeguarding ability towards carcinogenesis in this later aging phase, which is especially relevant in the skin, a tissue that is frequently exposed to mutagenic solar irradiation. The transition to phase 4 happens to coincide with epidemiological observations that pinpoint a strongly increasing risk of developing cancer from the chronological age range of 60 upwards [32]. Naturally further studies will be required to evaluate if any causal relationship between aging phase and cancer risk exists indeed, but the overlap in the chronological age ranges is intriguing and might warrant further investigations (Supplementary Figure 2D).

In summary, using multi-omics analysis we identified four aging phases in *ex vivo* human skin tissue of female participants over a wide age range. The phases appeared to be driven by actual biological age rather than chronological age, capturing distinct stages along the aging progression and allowed the data-driven reconstruction of the manifestation sequence postulated for the Hallmarks of Aging. Characterization of the mid- to late-life transition identified an extensive loss in pathway enrichment, with potential implications for life- and health-span in old age.

## MATERIALS AND METHODS

### Recruiting

The study was performed in agreement with the recommendations of the Declaration of Helsinki and all test subjects provided written, informed consent. Subjects were recruited in the age range of 20 to 80 years, with equal numbers of participants within each decade. Subjects were required to be female, in good health and belonging to phototypes II or III according to the Fitzpatrick scale [33], to limit non-age related variability in the data. Exclusion criteria included tattoos or scars in the test area, pigmentation disorders, pregnancy and medication such as anti-histamines or anti-inflammatory drugs within two weeks prior to study start. A detailed listing of exclusion criteria can be found in the Supplementary. Participants were further required to complete a self-assessment questionnaire on age, weight, height, smoker status, sun bathing habits, as well as food and drinking habits upon study start.

### Tissue sample preparation

The suction blister method applied in this study has been approved by the Ethics Commission of the University of Freiburg (general approval Dec 8, 2008; Beiersdorf AG No. 28857). Three suction blisters of 7 mm diameter were taken from the volar forearms of all subjects as previously described [34].

### Nucleic acid extraction

Tissue samples were suspended in the respective lysis buffers for DNA or RNA extraction and homogenized using an MM 301 bead mill (Retsch). DNA was then extracted using the QIAamp DNA Investigator Kit (Qiagen) according to manufacturer’s instructions. RNA was extracted using the RNeasy Fibrous Tissue Mini Kit (Qiagen) according to manufacturer’s instructions.

### Transcriptome sequencing

Transcriptome libraries were prepared using TruSeq Library Prep Kit (Illumina) and sequencing performed at 1x50 bp on Illumina’s HiSeq system to a final sequencing depth of 100 million reads per sample. Sequencing data was processed using a custom pipeline including Fastqc v0.11.7 [35] for quality control, Trimmomatic v0.36 [36] for trimming and Salmon v0.8.1 [37] for mapping and read quantification.

### Array based methylation profiling

Methylation profiling was performed using Illumina 450k (first time point) and EPIC (second time point) arrays. In order to ensure comparability of measurements, EPIC arrays were computationally reduced to include only probes present on the original 450k array using the minfi package [38] in R [39]. Methylation data was processed in minfi using the funnorm normalization method.

### Similarity network fusion and clustering

Prior to integration, the gene expression (log2 transformed transcripts per million) and CpG methylation data (M values) were batch corrected using the Combat algorithm [40] implemented in the sva package [41], following a feature selection step via filtering by median absolute deviation, retaining 10 % of the most informative features. The data was then integrated as previously described [18] using parameter settings of *k* = 10 (number of neighbors), *t* = 20 (number of iterations) and *alpha* = 0.5 (hyperparameter). Clustering on the fused network was performed via spectral clustering as previously described [18]. Measures used for the selection of cluster numbers were the eigen-gap statistic and rotation cost as proposed in the original method description [18], as well as visual inspection using heatmaps.

### Age clock analyses

Analyses of DNAm and transcriptomic age were performed as previously described [19]. DNAm age was calculated from M values, whereas transcriptomic age was predicted based on log2 transformed transcripts per million.

### Hallmark of aging gene sets

The HoA gene sets were generated from GO [42] and Reactome [43] gene sets by manually selecting matching pathways assigned to the nine Hallmarks of Aging [15]. A detailed list of genes annotated to each hallmark is provided in the Supplementary Material in .gmt format.

### Enrichment analyses

Enrichment analyses were performed using the PLAGE algorithm based on singular value decomposition as described in [44] and implemented in the GSVA [45] R package.

### Classification model to predict aging phase in longitudinal validation

To predict aging phase of re-invited subjects at the second time point, a random forest classifier was trained on the samples from the original cohort. Features were selected as the top 50 hits derived from differential gene expression analysis using DESeq2 [46] and differential methylation analysis using limma [47] from pairwise aging phase comparisons. The model was trained within the machine learning framework mlr [48], using the algorithm implemented in the original randomForest package [49]. Adjusted model hyperparameters included *ntree* = 1000 and $mtry\;=\;\sqrt{features}$. Accuracy of prediction was calculated as the area under the receiver operating characteristic curve (AUC) for multi-class comparisons, as implemented in the pROC package [50], and was derived from 5 x 5-fold repeated cross-validation.

### Pathway predictivity analysis

Pathway predictivity was assessed using random forest pathway classifiers, constructed using the gene sets generated in this study and using the Hallmark Process [20] gene sets downloaded from the Molecular Signatures Database v6.2 [51]. The models were trained by restricting the molecular data to that of genes annotated within a given hallmark and trained to predict the aging phase of every sample. Predictivity was determined as the accuracy of correct classification derived from 5 x 5-fold repeated cross-validation for each pathway model, giving insight on how well genes within the gene set allow a discrimination between the phases and was thus used as a measure of importance of the respective pathway. Samples were stratified with respect to the target variable in the cross-validation process in order to avoid unbalanced proportions in any fold that might lead to bloated accuracy measures. Hyperparameters of all models were adjusted to *ntree* = 1000 and $mtry\;=\;\sqrt{number\;of\;genes\;in\;pathway}$. To determine the predictivity of the HoA stratified for each of four aging phases, the classifiers were separately trained in a one-against-all type of setup, learning to distinguish a phase from all the others. Modeling parameters and cross-validation were chosen as described above, and results for the four phases were aggregated afterwards.

### General data analysis and visualization

Data analysis in R further included the usage of the package data.table [52], dplyr [53] and Hmisc [54] for data handling and general purpose functions, as well as the packages ggplot2 [55], ggpubr [56], ggsci [57], circlize [58] and pheatmap [59] for data visualization. Workflow diagrams were built using draw.io [60].

## Supplementary Material

Supplementary Materials

Supplementary Figures
