# Supplementary material for: Multi-omics network analysis reveals distinct stages in the human aging progression in epidermal tissue
Source: Aging (Albany NY). 2020 Jun 18;12(12):12393–409. doi: 10.18632/aging.103499 (PMC7343460; doi:10.18632/aging.103499)
Supplement: Supplementary Materials [file aging-12-103499-s001..pdf]

SUPPLEMENTARY MATERIALS

Recruiting appendix – inclusion and exclusion criteria

A detailed list of inclusion and exclusion criteria for the recruitment process is given below:

Inclusion criteria:

- Caucasian female subjects
- phototype II and III
- age: 20 to 85 years
- BMI between 18 and 25
- in good general health and mental condition
- healthy, intact skin on the test areas
- willing and capable to sign an informed consent document

Exclusion criteria:

- underweight or obesity (see BMI inclusion criteria)
- use of anti-histamines and corticoid-containing creams on the test sites in the last 4 weeks before the start of the study
- medication with anti-inflammatory or anti-coagulation agents (e.g. Ibuprofen, Aspirin, ASS, diuretics and thiazides, Marcumar) or antibiotics within 2 weeks prior to study start
- pregnancy, breast-feeding
- tattoos and/or scars on the test areas
- unusual scarring (e.g., hypertrophic scarring/ keloid formation)
- usage of self-tanning products within 14 days prior to study start
- pigmentation disorders
- abnormal reaction to sun
- hormonal diseases, not even in the past/childhood (diseases of the thyroid, growth disorders, hypo- or hyperfunction of the hypophysis, hormone replacement therapy, etc.)
- chronic (e.g. atopic dermatitis, psoriasis) or acute skin disease on the test sites
- physical and/or cosmetic treatments on the test sites within the last 14 days before the start of the study and/or during the study
- cancer in the past 10 years
- skin cancer generally
- severe disease (e.g. concerning cardiac / circulatory system, liver, kidney, lung), severe diabetes mellitus and/or chronic infectious disease (e.g. hepatitis, HIV)
- proven allergy to ingredients of cosmetics, patches, or intolerance reactions after the application of cosmetic products
- illness associated with fever (≥ 38.5°C) for at least 24 h within the last 7 days before the start of the study
