# Supplementary material for: Multi-omics network analysis reveals distinct stages in the human aging progression in epidermal tissue
Source: Aging (Albany NY). 2020 Jun 18;12(12):12393–409. doi: 10.18632/aging.103499 (PMC7343460; doi:10.18632/aging.103499)
Supplement: Supplementary Figures [file aging-12-103499-s002..pdf]

SUPPLEMENTARY FIGURES

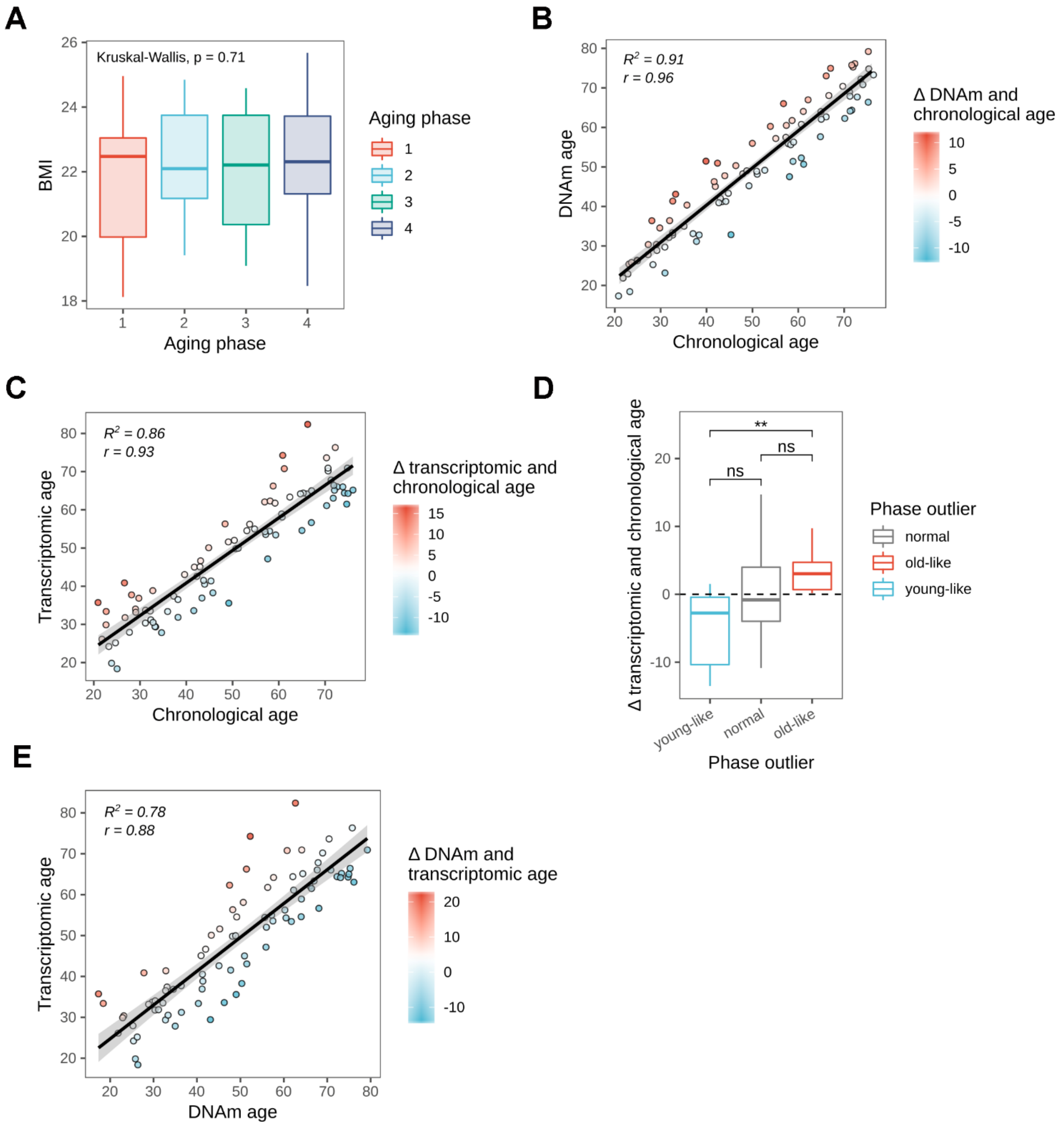

Supplementary Figure 1. (**A**) Boxplots showing the distribution of BMI within the identified aging phases, with no apparent bias detectable. (**B**) Relationship between chronological and DNAm age. (**C**) Relationship between chronological and transcriptomic age. (**D**) Boxplot showing the deviation of transcriptomic from chronological age based on aging phase outlier status. Statistical significance determined using pairwise T-tests. (**E**) Relationship between DNAm and transcriptomic age.

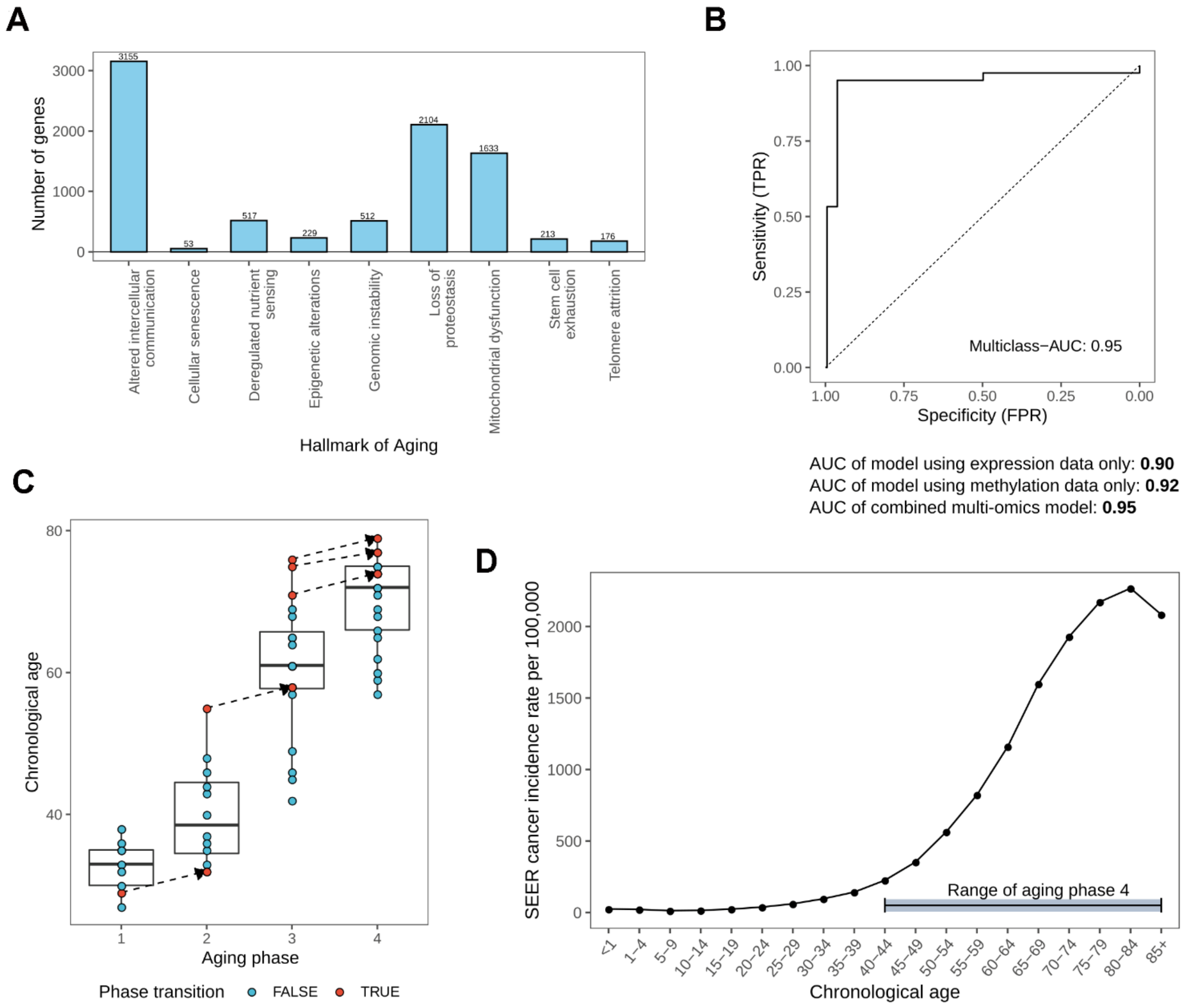

Supplementary Figure 2. (**A**) Sizes of the Hallmark of Aging gene sets generated from GO and Reactome pathways. (**B**) Receiver operating characteristic curve, showing the accuracy of classification of the random forest model trained on expression and methylation features to predict aging phases of the re-invited subjects at the longitudinal second time point. TPR and FPR rates were determined using 5 x 5-fold repeated cross-validation on the original data. (**C**) Classification of re-invited subjects into the respective aging phases, with transitioning subjects marked in red, distributed along their chronological age. Only re-invited subjects are shown. (**D**) Cancer incidence rate (aggregated SEER statistics from 1976-2016 for the United States of America), with the observed chronological age range of aging phase 4 in this study marked in blue.
